# The Therapeutic Value of Iodoform

**Published:** 1879-02

**Authors:** 


### Miscellaneous.

### The Therapeutic Value of Iodoform.

For many years the application of iodoform or teriodide of formyl has procured in the hands of Dr. J. Moleschott, of Turin (*Giornale della Reale Accademia di Torino*), effects so beneficial, that only the desire of studying physiologically the action of this valuable substance has been capable of restraining him from publishing his clinical experience.

It was in 1870, he says, that I was first induced to try iodoform in the case of a scrofulous man, aged about 30. In 1867 I had him several times under treatment for cold abscesses in the right groin and in the back part of the hip of the same side. In September of that year he suffered from swelling of the cervical glands on both sides, those on the left forming a uniform mass larger than a large fist. I treated the patient assiduously from September, 1870, to November, 1870. Externally, I applied iodide and bromide of potassium, iodated iodide, iodide and biniodide of mercury, chloride of ammonium, belladonna, cicuta and digitalis; internally, I gave iodide and bromide of potassium, iodized cod-liver oil, mineral waters rich in iodide (Sales water), without neglecting sea-baths. The success was very imperfect. It did not carry out the popular belief, often shared by medical men, that such tumours ought always to yield readily to the application of a simple iodine ointment. The case here referred to was one of the many obstinate ones. The advantages gained after a course of treatment diligently carried out for over three years, still left much to be desired. The young man was desirous of being married; but his friends aways ridiculed the proposal on account of the deformity caused by the tumour. Stimulated by the desire to free him from this, I searched through jonrnals and books, and met with praises of iodoform; but I regret that I do not remember which author first led me to entertain hope. I prescribed one part of iodoform and fifteen of elastic collodion, to be applied by a brush night and morning. This treatment was commenced November 20, 1870; and on December 18th the tumour was reduced to one-half its size; on February 2, 1871, it had almost disappeared; and when I saw him on April 5th no trace of it could be seen. The patient was then suffering from vesical catarrh, gingivitus, and a little palpitation; and in the next September he had perityphlitis. In March, 1871, when the tumour had nearly disappeared, the urine contained albumen for a short time. When he last came to consult me, in October, 1877, there had been no return of the glandular swellings, or of the cold abscesses. This case did not remain singular in the circle of my practice. Two little daughters of a schoolmaster, aged respectively 8 and 10 years, had for a year or more glandular swellings in the neck, of the size of a hen's egg. Ointment of iodide of potassium failed. With iodoform, applied in the manner above described, they were cured in a few weeks.

Of many simular cases, in all of which iodoform subdued that which had obstinately resisted iodide of potassium, I relate one which appears remarkable, since the cartilaginous hardness of the tumour had led me to doubt the efficacy of the remedy in which I already placed much trust. A chambermaid had on the right side of her neck a tumour as large as a middle-sized hen's egg, and as hard as cartilage. She had been disfigured by it for many years; and, although it was not painful, she desired its removal. I applied iodoform in the form of ointment (1 in 15). At the end of three months a very favorable effect was produced; she assiduously followed up the treatment, and within a year the swelling had disappeared.

The last case of this kind which I saw was in a man who had in his right inguinal region a large mass of swollen glands. The tumour, which had commenced with pain, had been treated by a medical man with poultices and incisions. But, in spite of the continued suppuration, the groin remained swollen to such an extent as almost to disable the patient from the discharge of his duties. When this condition had lasted three months he asked my advice. In four weeks, iodoform ointment effected a complete cure.

This favorable experience of the efficacy of iodoform in combating swellings of the lymphatic glands, was crowned by the improvement obtained in a case of splenic leuchæmia. The subject was a lady whose spleen was doubled in size, and could be easily felt by the hand. Her blood contained one white corpuscle to fifty red, in place of the usual proportion of one to 357. The principal symptoms were prostration, pallor, obstinate diarrhœa, especially severe at the menstrual periods, and a great tendency to acute painful œdema. There was no hemorrhage of the lymphatic glands. On the other hand, the patient had two attacks of severe pain in the sacrum and last lumbar vertebræ, probably dependent on the participation of the marrow of the bones in the disease. The case might then be considered as one of splenic and myelogenous leuchæmia, but remarkable for its severity and long duration. When she first came under my care in January, 1870, she had for several months remained in bed. She did not tolerate quinine, nor iron, nor any other metallic remedy. On the other hand, she obtained advantage from aromatic baths of 26 to 27 Reaumur (90.5 to 92.75 Fahr.), continued for not more than three minutes, and from painting over the region of the spleen with iodoformized collodion; this treatment was commenced in 1871. The diarrhœa required appropriate treatment from time to time. Fortunately, the patient's appetite never failed; she could digest venison and other nutritious food. The swelling of the spleen returned several times, but was always restrained by the external application of iodoform. The proportion between the two kinds of blood corpuscles had for some time been normal.

I do not by any means assert that the remedy for leuchæmia has been found in iodoform. The cure is not sufficiently complete, nor the case severe enough; and I have not had an opportunity of trying the same treatment in other cases; but the result of this first attempt seems to encourage further trial.

From the time when I verified the solvent effect of iodoform, I applied it repeatedly in the treatment of swollen and indurated inguinal glands of syphilis. In these cases I gave protoiodide of mercury according to Simon's excellent method, or iodoform in pills, in doses varying from 5 to 10 centigrammes ($\frac{3}{4}$ to $1\frac{1}{2}$ grains) in the day; its effect were so salutary, that I can warmly recommend iodoform in the treatment of syphilis.

Judging from the related facts, iodoform, before promoting absorption, should determine the destruction of the primitive elements. This may be said to be its *modus operandi* in orchitis, in which I have several times obtained resolution in a period varying from five to eight days, by the application of iodoformized collodion.

In cases of effusion into serous cavities, iodoform has suppassed my expectation. By painting with the iodoform dissolved in elastic collodion, I have seen fluid dispersed which had collected in the pluera, pericardium, and peritoneum, and beneath the arachnoid.

In the case of a lady, wife of a well-known officer, who suffered from insufficiency of the tricuspid valve, I twice obtained absorption of a dangerous pericardial effusion.

At Nervi, some years ago, a gentleman, aged 45, the subject of pulmonary tuberculosis, had ascites to such an extent that he could only remain in a semi-recumbent position on his back, and could not bend his body. I thought that paracentesis would be required, in spite of the anæmic state of the body. I determined, however, to try iodoformized collodion, giving at the same time diuretic pills. The collection of fluid disappeared in about fifteen days, with an abundant discharge of urine; and it did not return during the remaining year and a half of the patient's life. From that time, I have made it a rule not to advise paracentesis, without having first tried the external application of iodoform. I confess that it was not always efficacious. We cannot be surprised at this, since in many cases we cannot eliminate the cause of exudation of the fluid, we cannot prevent it from again collecting, and the obstacle to the circulation may be so great that no treatment succeeds in stimulating absorption.

Of all the satisfactory results which I have obtained from the application of iodoform, the greatest has been in the acute hydrocephalus of children. In recent years, I have reported three complete cures among five cases of this fatal disease, two of which appeared to be in a truly desperate condition. The fixed look, the lost senses, the sopor, the convulsions, the sunken abdomen, the vomiting, the unfrequent pulse, the dilated and unequal pupils, the tonic contraction of the cervical muscles, completed a picture which could be easily recognized. I ordered iodoform, dissolved in collodion, or in the form of ointment, to be applied three or four times daily to the cervical region, and over the mastoid processes. the forehead and the temples. I must not omit to state that the children at the same time had small doses of calomel, and purgative clysters.

I will here mention a case of prepatellar cystic hygroma. The subject was a valet in a large, who, having to keep polished the furniture in the rooms, was obliged often to kneel on his right knee. With paintings of iodoformized collodion, the swelling of the bursa in front of the patella was reduced in fifteen days, although it had already existed as many months.

In chronic arthritis, also, I have had much reason to praise iodoform. Two cases are particularly memorable. One was that of a little giri nine years old, daughter of a teacher of swimming, who, in April, 1875, when I undertook to treat her, had been suffering for nine months with inflammation of the left knee. The suppuration was very diffuse, and the child suffered severe pain and was very much weakened. The use of iodoform dissolved in collodion, of iodide of iron in the form of Blancard's pills, and absolute rest, so far restored her that in May, 1876, only a slight stiffness of the joint remained.

The other case was one of fungous inflammation of the articulation of the left foot in a boy aged 15. He had been confined to bed many months, and his parents had no further hope. Two very skillful surgeons had declared that there was no resource but amputation; but, his parents not consenting, the patient was removed from the hospital. The left tarsus was twice as large as right, and was surrounded by eight or nine suppurating and fungating sores; in more than one spot, the suppuration reached the bone. There was no pain. I had iodoformized collodion applied twice daily to all parts of the foot where the skin remained sound, and the ulcers were first treated with chamomile baths, and afterwards with solutions of nitrate of silver (2 to 10 per cent.); iodide of iron was given internally. At the end of a year, the boy could walk, the sores were all healed, the tarsus was scarcely swollen, and all movements were possible, though less free than in the other foot. He had no return of the disease, although he committed several imprudent acts. At present he is able to work; and his parents who had resigned themselves to seeing him perish, rejoice in the possession of a robust lad.

From all that has been said above, iodoform appears to be a remedy which has a powerful resolvent action, and causes the absorption of formative elements and of collections of exuded fluid. But to these effects it unites the valuable property of assuaging pain. This may be proved in attacks of gout. I have often succeeded in removing or in considerably relieving the most severe pain and other inflammatory symptoms of gout, within twenty-four hours, by painting the parts with iodoformized collodion.

Less certain is the success of iodoform in chronic rheumatism affecting several joints, and I have found it quite unreliable as a remedy against the pain of acute articular rheumatism.

As a sedative remedy, I have used iodoform in a large number of cases of neuralgia, mostly intercostal, cardiac, sciatic, and articular. I have most frequently applied it dissolved in collodion, but have also used it in the form of ointment.

In one case, intercostal neuralgia was accompanied with syphilitic myocarditus, without disease of the valves. The patient, a merchant at Alba, was affected on the slightest movement, even a short walk, with giddiness and spasmodic pain in the region of the heart. He was cured by the internal and external use of iodoform; but he had to continue the treatment with short interruptions for several months. He took internally from 5 to 10 centigrammes in twenty-four hours, in the form of pill.

Patients very often present themselves, conplaining of intercostal pain in the heart, radiating towards the left clavicle. Such persons not unfrequently feel palpitation, and fear that they have disease of the heart, although they are quite free from it. These patients are comforted if an opportune treatment free them from their pains; and the external application of iodoform fulfils this object admirably. Along with the pains, the unfounded dread of cardiac disease disappears.

Although desirous to avoid making a complete enumeration of the services which iodoform has rendered me, I must make brief mention of a case in which I cured a true neurttis following typhoid fever in a young man, and affecting the trunk of the left sciatic nerve. When the patient sought my help, in the autumn of 1870, his sufferings had lasted several days. The slightest pressure on the nerve caused intolerable pain; and the patient, who in other respects might be called convalescent, was obliged to remain motionless in bed. The pain was very soon relieved by iodoform; but the leg remained so weak, that for several weeks the patient walked on crutches: and he did not recover perfectly until a prolonged stay at Nervi.

A remedy which combines in itself antiphlogistic, resolvent, and sedative properties; which embraces a field of action extending from neuritis to neuralgia, from leuchæmia to tuberculous meningitis, from lymphoma to collections in serous bursæ, from attacks of gout to hygroma; a remedy which in the multiplicity and energy of its attacks competes with quinine and with cold water, might be regarded as miraculous, if it had not its defects like every other good thing. Fortunately, however, these defects will not be very detrimental to the service which it is capable of rendering to suffering humanity.

The most important defect in iodoform is its perceptible odour, which is much more preceptible when used with collodion than when applied in the form of ointment. Its internal use is sometimes followed by disagreeable eructations. It cannot be said that the odour is repulsive; it is rather oppressive. In the collodion solution, it reminds me of a photographer's labratory. In order to overcome the objections arising from this odour, the following rules should be followed.

The box of iodoform ointment, or the bottle of iodoformized collodion, should be kept at a distance from the window, in a well-closed tin case; this retards the decomposition of the iodoform, which goes on rapidly in the light. The surface to which it is applied should be covered by a thin layer of gutta percha. Finally, unless its action be urgently required, the application should be made only in the evening; preference being given to the ointment, which in the morning can be easily washed off with a little soap and water, so as to leave no smell.

Another defect of iodoform is, that it sometimes causes palpitation. I have as yet not often observed this; but it repeatedly occurred in a hysterical lady for whom I prescribed iodoform internally as a remedy for hemicrania. This defect, causes me to recognize a conspicuous advantage, which I desire to see confirmed by later experience.

Some months ago, I had under my care a lady, wife of a professor of literature, suffering from mitral insufficiency without compensating hypertrophy of the left ventricle. Irregularity of the heart-beat was the most troublesome symptom of the exhaustion of the cardiac muscle in its attempts to overcome the obstacle. She had *malaise*, diminution of the urine, nervous attacks, oppression, and dyspnœa. The radial pulse was often scarcely perceptible, Very small doses of digitalis (30 or 40 centigrammes in infusion. in twenty-four hours) several times produced a sensible improvement; but the stomach did not bear it well, and it therefore became necessary to suspend the use of the remedy before a satisfactory advantage had been obtained. Remembering the experience referred to above, I had recourse to iodoform. which I prescribed in doses of 6 or 7 centigrammes (about 0.9 to 1 grain daily) in form of pills. The patient had scarcely taken it two days, when I found the heart's action regular and the radial pulse well developed. The heart, which seemed to have given up all rhythm, had regained a regular beat. The same success was repeated several times in this patient, at intervals of various length.

This and similar observation justify me in asking whether iodoform, administered internally in daily doses of 5 to 10 centigrammes. may not compete with digitalis—I mean those small doses of digitalis which render the action of the heart stronger and more regular.

Iodine is found in the urine after the external and internal use of iodoform, but rather more slowly after the former than after the latter. In either case, the complete elimination of the medicine requires much time, so that traces of iodine may be found in the urine at the end of four or five days.

Allied in constitution to chloform, iodoform is in many cases a valuable narcotic; but to the quality of relieving pain it adds in a high degree the effects of a powerful preparation of iodine. Neither iodide of potassium nor pure iodine can be compared with iodoform, when we consider its efficacy as a promoter of resolution and absorption of tumours and exudations. It seems probable that the surprising effects of iodoform are to be attributed to the facility with which iodine is liberated from it, so as to act in a nascent state on the elements of the organism.

Notwithstanding the inconveniences which I have not wished to conceal, I dare promise for this remedy a great future.—*London Med. Record*, Nov. 15, 1878.—*Monthly Abstract Medical Sciences.*

